# Gene- gene interaction between *PPARG* and *CYP1A1* gene on coronary artery disease in the Chinese Han population

**DOI:** 10.18632/oncotarget.16186

**Published:** 2017-03-14

**Authors:** Xiaojiang Zhang, Shuzheng Lv, Chengjun Guo, Conghong Shi, Yunpeng Chi, Lin Zhao, Guozhong Wang, Zhisheng Wang

**Affiliations:** ^1^ Department of Cardiology, Beijing Anzhen Hospital, Capital University of Medical Sciences, Beijing 100029, China; ^2^ Baotou Fourth Hospital, Baotou, Inner Mongolia, 014030, China

**Keywords:** PPARG, CYP1A1, coronary artery disease, SNP, interaction

## Abstract

**Aims:**

To observe the influence of the peroxisome proliferator-activator receptor-G (*PPAR-G*) gene and cytochrome P4501A1 (*CYP1A1*) single-nucleotide polymorphisms (SNPs), and interactions among several SNPs on coronary artery disease (CAD) risk.

**Methods:**

A total of 1106 participants (including 583 males and 523 females) including 550 CAD patients and 556 control subjects were recruited in this study, and the mean age for these participants was 55.5 ± 11.8 years old. Logistic regression was used to observe association of SNP within PPARG and CYP1A1 with CAD risk and GMDR model was used to screen the best interaction combinations.

**Results:**

CAD susceptibility was higher in those with homozygous mutant of rs10865710, rs1805192 and rs4646903 than those with wild-type homozygotes, OR (95%CI) were 1.47 (1.15–1.92), 1.69 (1.27–2.09) and 1.72 (1.35–2.32), respectively. We also found a significant two-locus model involving rs1805192 and rs4646903 (*p* = 0.0107), and the cross-validation consistency of this locus model was 10 of 10, the testing accuracy of this model is 62.17%. Logistic regression shown that CAD risk was the highest in those with rs1805192- Pro/Ala or Ala/Ala and rs4646903- AG+GG genotype, and was lowest in those with rs1805192- Pro/ Pro and rs4646903- AA genotype, OR(95%CI) = 3.56 (1.91–5.42).

**Conclusions:**

Polymorphism in rs10865710, rs1805192 and rs4646903 and interaction between rs1805192 and rs4646903 were related with increased CAD susceptibility.

## INTRODUCTION

Coronary artery disease (CAD), which was an important chronic disease cause of death worldwide [1], has accounts to almost 40% of all mortalities in many countries [2, 3]. CAD is influenced by multifactorial factors and may result from the complex synergistic reaction between genetic background and environmental factors [4]. Previously several CAD related variants and environmental factors were reported, such as type 2 diabetes mellitus (T2DM), dyslipidemia, hypertension and genetic factors, including proliferator-activator receptor-G (*PPAR-G*) and Cytochrome P450 (*CYP*) family, are significant risk factors for CAD [5–7].

The *PPARG* gene, located at 3p25-24, and plays a non-ignorable role in adipocytes differentiation, insulin sensitivity regulation, and its variation has been reported association with some CAD related risk factors, such as T2DM or metabolic syndrome (MS) [8]. Additionally, *PPARG* also play important role in regulation for fatty acid metabolism, perhaps in adipose tissue storage and free fatty acids reduction. Many studies have been conducted to investigate the association of *PPARG* polymorphism with CAD susceptibility, but the results obtained from these studies were controversial [9–11]. *CYP* is a kind of enzymes, which could mediate the oxidative metabolism of exogenous and endogenous molecules [12], and metabolism for several endogenous molecules, such as cholesterol, estrogens, androgens, and so on [13, 14]. Recently, several studies have reported the association between *CYP1A1* polymorphisms and the risk of CAD susceptibility [15–17]. As fore- mentioned that CAD risk was influenced by many gene polymorphisms or interactions among several genes, considering *CYP1A1* and *PPARG* both are risk factor of CAD, however, to date, less study focused on *PPAR G- CYP1A1* interaction on CAD risk was reported, so the aim of this study was to investigate the impact of *PPARG* and *CYP1A1* polymorphisms, and synergistic interaction between the two genes on CAD risk.

## RESULTS

In this study, 1106 participants (583 men, 523 women) including 550 CAD cases and 556 control subjects were recruited, and the mean of age for these participants is 55.5 ± 11.8 years old. Table 1 shows the clinical characteristics for the participants in cases and controls. The means of BMI and WC are higher in CAD case group than that in control group. There are no significant different between cases and controls in distribution of males, rate of high- fat and low fiber diet, alcohol drinking and smoking and mean of age.

**Table 1:** General characteristics of study participants in CAD cases and controls

Variables	CAD cases (*n* = 550)	Controls (*n* = 556)	*p-*values
Age (years)	57.1 ± 12.8	56.9 ± 13.0	0.780
Males N (%)	287(52.2)	296(53.2)	0.725
Drinking N (%)	231(42.0)	212(38.1)	0.189
Smoke N (%)	202 (36.7)	190(34.2)	0.374
WC(cm)	85.1 ± 14.1	82.3 ± 15.3	0.002
BMI(kg/m^2^)	24.7 ± 9.8	23.4 ± 9.5	0.025
High fat diet N (%)	120(21.8)	105 (18.9)	0.226
Low fiber diet N (%)	138 (25.1)	116(20.9)	0.095

Abbreviations: WC, waist circumference; BMI, body mass index

Table 2 shows the frequencies of alleles and genotypes within four SNPs in cases and controls. We found that the variants in rs10865710, rs1805192 and rs4646903 were related with increased CAD risk after covariant adjustment. CAD risks were higher in carriers of homozygous mutant of rs10865710, rs1805192 and rs4646903, and lower in those with wild-type homozygotes, OR (95%CI) were 1.47 (1.15–1.92), 1.69 (1.27–2.09) and 1.72 (1.35–2.32), respectively.

**Table 2:** Analysis on the association between 4 SNPs and CAD risk

SNPs	Genotypes and Alleles	Frequencies N (%)		OR(95%CI)*	*P-* values	H-W test
		Cases (*n* = 550)	Controls(*n* = 556)			
PPAR G rs10865710						
	CC	287(52.2)	335(60.3)	1.00		0.351
	CG	223(40.5)	198(35.6)	1.22(1.06–1.47)	0.018	
	GG	40(7.3)	23(4.1)	1.86(1.42–2.34)	< 0.001	
	GG+CG	263(47.8)	221(39.7)	1.47(1.15–1.92)	< 0.001	
	C	797(72.5)	868(78.1)			
	G	303(27.5)	244(21.9)			
rs1805192						
	Pro/Pro	283(51.4)	340(61.2)	1.00		0.620
	Pro/Ala	211(38.4)	187 (33.6)	1.43(1.12–1.78)	< 0.001	
	Ala/Ala	56(10.2)	29(5.2)	2.15(1.56–2.86)	< 0.001	
	Ala/Ala+ Pro/Ala	267(48.6)	216(38.8)	1.69(1.27–2.09)	< 0.001	
	Pro	777(70.6)	867(78.0)			
	Ala	323(29.4)	245(22.0)			
CYP1A1 rs4646903						
	TT	270(49.1)	350(63.0)	1.00		0.880
	TC	219(39.8)	183(32.9)	1.52(1.24–1.97)	< 0.001	
	CC	61(11.1)	23(4.1)	2.08(1.44–2.72)	< 0.001	
	TC+CC	280(50.9)	206(37.0)	1.72(1.35–2.32)	< 0.001	
	T	759(69.0)	883(79.4)			
	C	341(31.0)	229(20.6)			
rs1048943						
	AA	310(56.4)	337(60.6)	1.00		0.889
	AG	199(36.2)	191(34.4)	1.12(0.94–1.47)	0.428	
	GG	41(7.4)	28(5.0)	1.38(0.90–1.95)	0.625	
	GG +AG	240(43.6)	219(39.4)	1.18(0.93–1.62)	0.516	
	A	819(74.5)	865(77.8)			
	G	281(25.5)	247(22.2)			

^a^ Adjustment for gender, age, alcohol consumption, high fat diet, low fiber diet, BMI and WC.

GMDR model was used to screen the potential best interaction combination among SNPs within *PPARG* and *CYP1A1*. In Table 3, we found that there was a significant gene–gene interaction between rs1805192 and rs4646903. In this model, the cross-validation consistency is 10/10 and the testing accuracy is 62.17%. Logistic regression indicated that participants with rs1805192- Pro/Ala or Ala /Ala and rs4646903- TC+CC genotype have the highest CAD risk, compared to participants with rs1805192- Pro/ Pro and rs4646903- TT genotype, OR (95%CI) was 3.56 (1.91–5.42), after covariant adjustment (Table 4).

**Table 3:** Best gene–gene interaction models, as identified by GMDR

Locus no.	Best combination	Cross-validation consistency	Testing accuracy	*p*- values ^a^
2	rs1805192 rs4646903	10/10	0.6217	0.0010
3	rs1805192 rs4646903 rs10865710	8/10	0.5399	0.0547
4	rs1805192 rs4646903 rs10865710 rs1048943	7/10	0.4958	0.1719

^a^ Adjustment for gender, age, alcohol consumption, high fat diet, low fiber diet, BMI and WC.

**Table 4:** Interaction between rs1805192 and rs4646903 on CAD risk

rs1805192	rs4646903	OR (95% CI) ^a^	*P*-values
PP	TT	1.00	-
PP	TC+CC	1.56 (1.19–2.04)	0.001
PA or AA	TT	1.37 (1.06–1.83)	0.032
PA or AA	TC+CC	3.56 (1.91–5.42)	< 0.001

^a^ Adjustment for gender, age, alcohol consumption, high fat diet, low fiber diet, BMI and WC.

Abbreviations: P: Pro, A: Ala

Pairwise LD analysis between SNPs was performed and the D′ value between rs10865710 and rs1805192 was 0.835, and the D′ value between rs4646903 and rs1048943 was 0.808. So we also conducted haplotype analysis between rs10865710 and rs10865710, between rs4646903 and rs1048943. We found a haplotype containing the rs10865710-G and rs1805192-A alleles within *PPARG* were associated with a statistically increased CAD risk, OR (95%CI) = 2.08 (1.47–2.72), *P* < 0.001, however we did not find any haplotype combination within *CYP1A1* associated with CAD risk (Table 5).

**Table 5:** Haplotype analysis on association of PPARG and CYP1A1 gene and CAD risk

Haplotypes	SNP1	SNP2	Frequencies		OR (95%CI)	*p*-values*
			Case group	Control group		
*PPARG*	rs10865710	rs1805192				
H1	C	P	0.4701	0.5467	1.00	--
H2	G	P	0.2167	0.2131	1.16 (0.82–1.69)	0.670
H3	C	A	0.2015	0.1971	1.29 (0.93–1.78)	0.412
H4	G	A	0.1117	0.0431	2.08 (1.47–2.72)	< 0.001
*CYP1A1*	rs4646903	rs1048943				
H1	T	A	0.5322	0.5431	1.00	--
H2	C	A	0.2064	0.2101	1.06 (0.72–1.48)	0.562
H3	T	G	0.1897	0.1947	0.98 (0.67–1.43)	0.635
H4	C	G	0.0717	0.0521	1.23 (0.77–1.81)	0.724

*Adjusted for gender, age, smoking and BMI

## DISCUSSION

In current study based on Chinese Han population, we found that variants in rs10865710, rs1805192 and rs4646903 were associated with higher CAD risk. The *PPARG* gene plays an important role in adipocytes differentiation, insulin sensitivity regulation, and its variation has been reported association with some CAD related risk factors, such as T2DM or metabolic syndrome (MS) [8]. Additionally, *PPARG* also play important role in regulation for fatty acid metabolism, perhaps in adipose tissue storage and circulating concentrations of free fatty acids reduction. Many studies have been conducted to investigate the association between *PPARG* polymorphism and CAD risk, but the results obtained from these studies were controversial [9–11]. Rhee et al. [9] suggested that rs1805192 polymorphism in exon B of PPARG was not associated with prevalence of CAD in Korean adults, the similar results were also found in Caucasians [18] and in Indian Population [19]. In a Chinese study, Zhou et al. [10] also reported no association was obtained between and HDL cholesterol in CAD patients. In gender difference analysis, the rate for the T allele is significantly lower in males, subjects with age less than 62 years, and non-smokers. But Liu et al. [20] suggested that both rs1805192 and rs10865710 polymorphisms were associated with CVD related risk factors, but was not associated with lipid and nutrition metabolism. Wu et al. [21] performed a meta- analysis and indicated that the Ala allele in rs1805192 might related to increased CAD risk, but this effect is stronger in Caucasians and barely in Asians.

CYP is a kind of enzymes, which could mediate the oxidative metabolism of exogenous and endogenous molecules [12], could also play an important role in metabolism for several endogenous molecules, such as cholesterol, estrogens, androgens, and so on [13, 14]. Recently, several studies have reported the association between *CYP1A1* polymorphisms and the risk of CAD susceptibility [15–17]. Manfredi et al. [22] found that *CYP1A1* polymorphisms did not influence CAD susceptibility. Taspinar et al. [23] also suggested that *CYP1A1* genotypes were not significantly different between patients and controls. A meta- analysis [24] suggested that the *CYP1A1* rs4646903 polymorphism was not correlated with CAD risk. Yeh et al. [25] suggested that *CYP1A1* polymorphism may be associated with the lower susceptibility to CAD, particularly in non-smokers. However, some studies concluded different results. Sultana et al. [26] suggested that ischemic stroke (IS) risk was higher in subjects with *CYP1A1*- CC genotype. Wang et al. [15] reported that *CYP1A1 MspI* polymorphisms are associated with increased CAD risk. Zou et al. [17] conducted a case- control study for Chinese Uygur and Han and they indicated that both rs12441817 and rs4886605 within *CYP1A1* gene are correlated with CAD susceptibility.

CAD susceptibility is influenced by many gene polymorphisms and gene- gene interactions, considering *CYP1A1* and *PPARG* both are risk factor of CAD, however, till now, no study focused on *PPARG- CYP1A1* interaction on CAD risk was reported. In this study, we found that interaction between rs1805192 and rs4646903 was also correlated with CAD risk, and the CAD risk was highest in participants with rs1805192- Pro/ Ala or Ala/ Ala and rs4646903- TC+CC genotype, and was lowest in participants with rs1805192- Pro/ Pro and rs4646903- TT genotype. The potential mechanism for this interaction was not very clearly, some studies have reported that both *PPARG* and *CYP1A1* gene polymorphisms were associated with CAD related- risk factors, such as T2DM, obesity and hypertension, and so on. Maybe this combined or crossover effect could lead to the interaction between *PPARG* and *CYP1A1* gene on CAD risk.

There are several limitations in our study. Firstly, more SNPs within *PPARG* or *CYP1A1* gene should been studied in the future study, particularly some less studied SNPs. Secondly, some environmental risk factors should be included to investigate gene- environment interaction, such as smoking or alcohol drinking and so on.

In conclusion, we found that variants in rs10865710, rs1805192 and rs4646903 were significantly related with increased CAD risk. We also found a significant interaction between rs1805192 and rs4646903, and CAD risk was highest in participants with rs1805192- Pro/ Ala or Ala/ Ala and rs4646903- TC+CC genotype, and was lowest in those with rs1805192- Pro/ Pro and rs4646903- TT genotype.

## MATERIALS AND METHODS

### Subjects

**Figure 1:**
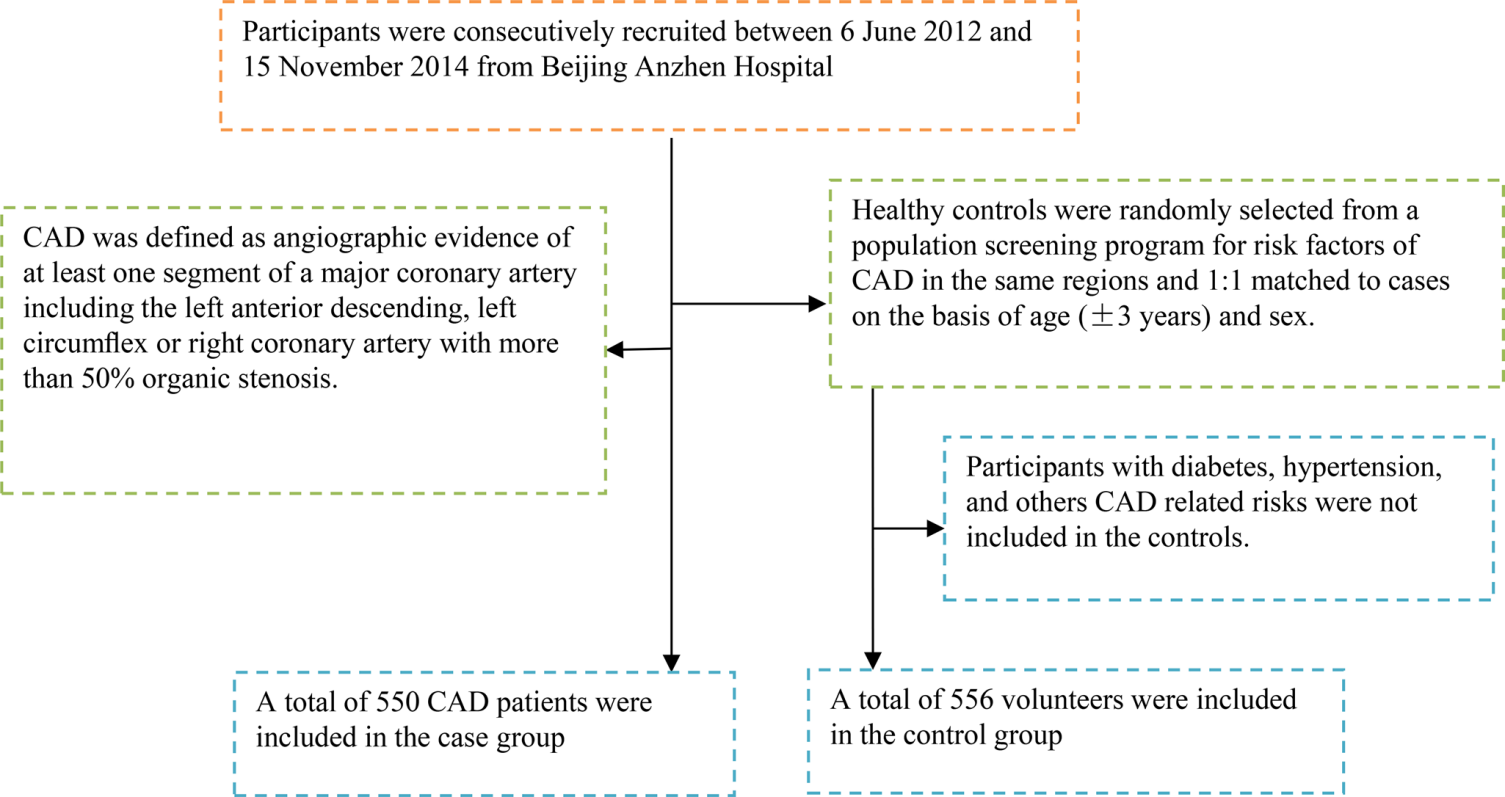
A flowchart on study population selection and exclusion

All participants were recruited from 6 June 2012 to 15 November 2014 from Beijing Anzhen Hospital. The CAD patients were diagnosed by coronary angiography using a quantitative coronary angiographic system [27]. The control subjects were randomly recruited from another population investigation program for chronic disease and related risk factors in our city and with nearly 1:1 matched to cases group on the basis of age (± 3 years) and gender. Participants with hypertension, type 2 diabetes (T2DM), and others CAD related risks were excluded from the control group (Figure 1). Writing informed consents were signed by all participants.

### Information collection

We collected related information by using questionnaire and body measurement. In the questionnaire investigation, some information, such as demographic information, alcohol drinking, tobacco smoke and diet habit information for all cases and controls were collected. In the body measurement procedure, some parameters, such as waist circumference (WC), body weight and height were measured, and then BMI was calculated. Currently alcohol drinkers were defined as those who drink more than 1 times every month; those who have smoked for at least 100 cigarettes and still smoked at the time of the investigation were considered as current smokers. Blood samples of all participants were all collected during the investigation.

### Genomic DNA extraction and genotyping

We selected SNPs within the *PPARG* and *CYP1A1* gene according to the following methods, including: 1) which have been reported associations with CAD or risk factors of CAD; 2) minor allele frequency (MAF) greater than 5%. Taking into account the limitations of human, material and financial resources, a total of two SNPs in *PPARG* and two SNPs in *CYP1A1* were selected for genotyping in the study: rs1805192, rs4646903, rs10865710 and rs1048943. Genomic DNA of all participants was extracted from the collected EDTA-treated whole blood by using the DNA Blood Mini Kit (Qiagen, Hilden, Germany) according to the instruction manual. The genotyping for all SNPs were performed by using Taqman fluorescence probe. Table 6 shows the corresponding probe sequences and description for all SNPs. ABI Prism7000 software was used for genotyping. A 25 μl reaction mixture including 1.25 ul SNP Genotyping Assays (20×), 12.5 μl Genotyping Master Mix (2×), 20 ng DNA, and the conditions were as follows: initial denaturation for 9 min and 94°C, denaturation for 18 s and 93°C, annealing and extension for 80 s and 62°C, 50 cycles.

**Table 6:** Description and Probe sequence used for Taqman fluorescence probe analysis for 4 SNPs

ID	SNP	Chromosome	Functional Consequence	Major/minor allele	Probe sequence
*PPARG*					
rs10865710	*C681G*	3	Exon_A2	C/G	5′-TTGGCATTAGATGCTGTTTTGTCTT[C/G] ATGGAAAATACAGCTATTCTAGGAT-3′
rs1805192	*Pro12Ala*	3	Exon_B	C/G	5′-ACCTCAGACAGATTGTCACGGAACA[C/T] GTGCAGCTACTGCAGGTGATCAAGA-3′
*CYP1A1*					
rs1048943	*A4889G*	15	Missense	A/G	5′-CAAGCGGAAGTGTATCGGTGAGACC[A/G] TTGCCCGCTGGGAGGTCTTTCTCTT-3′
rs4646903	*T6235C*	15	Downstream variant 500B	T/C	5′- TTGTTTCACTGTAACCTCCACCTCC[C/T] GGGCTCACACGATTCTCCCACCTCA-3′

### Statistical analysis

The means and standard deviations (SDs) were calculated for normally distributed continuous variables and were compared between cases and controls using Student's *t* test, and percentages are also calculated for categorical variables and are compared between case group and control group by using χ^2^ test. The association between SNPs and CAD and Hardy-Weinberg equilibrium (HWE) were performed by using SNPStats. Logistic regression was used to observe association of SNP within *PPARG* and *CYP1A1* with CAD risk. Generalized multifactor dimensionality reduction (GMDR) model was used to analyze the gene- gene interaction, some parameters including cross-validation consistency, the testing balanced accuracy and the sign test were calculated, a sign test or a permutation test (providing empirical *p*-values) for prediction accuracy can be used to measure the significance of an identified model.
